# Self-Nanoemulsifying Drug Delivery Systems Containing *Plantago lanceolata*—An Assessment of Their Antioxidant and Antiinflammatory Effects

**DOI:** 10.3390/molecules22101773

**Published:** 2017-10-20

**Authors:** Azin Kalantari, Dóra Kósa, Dániel Nemes, Zoltán Ujhelyi, Pálma Fehér, Miklós Vecsernyés, Judit Váradi, Ferenc Fenyvesi, Ákos Kuki, Sándor Gonda, Gábor Vasas, Rudolf Gesztelyi, Anayatollah Salimi, Ildikó Bácskay

**Affiliations:** 1 Department of Pharmaceutical Technology (www.pharm.unideb.hu), University of Debrecen, Nagyerdei körút 98, 4032 Debrecen, Hungary; azin.kalantari@pharm.unideb.hu (A.K.); kdorik98@gmail.com (D.K.); nemes.daniel@pharm.unideb.hu (D.N.); ujhelyi.zoltan@pharm.unideb.hu (Z.U.); feher.palma@pharm.unideb.hu (P.F.); vecsernyes.miklos@pharm.unideb.hu (M.V.); varadi.judit@pharm.unideb.hu (J.V.); fenyvesi.ferenc@pharm.unideb.hu (F.F.); 2 Department of Applied Chemistry (www.pharm.unideb.hu), University of Debrecen, Nagyerdei körút 98, 4032 Debrecen, Hungary; kuki.akos@science.unideb.hu; 3 Department of Pharmacognosy (www.pharm.unideb.hu), University of Debrecen, Nagyerdei körút 98, 4032 Debrecen, Hungary; gonda.sandor@science.unideb.hu (S.G.), vasas.gabor@pharm.unideb.hu (G.V.); 4 Department of Pharmacology (www.med.unideb.hu), University of Debrecen, Nagyerdei körút 98, 4032 Debrecen, Hungary; gesztelyi.rudolf@med.unideb.hu; 5 Nanotechnology Research Center, Ahvaz Jundishapur University of Medical Sciences, Ahvaz 61357-33184, Iran; anayatsalimi2003@yahoo.com

**Keywords:** SNEDDS, *Plantago lanceolata*, antioxidant and anti-inflammatory effects, DPPH test, cytotoxicity investigation, MTT-test, Caco-2 cells

## Abstract

The most important components of *Plantago lanceolata* L. leaves are catalpol, aucubin, and acteoside (=verbascoside). These bioactive compounds possess different pharmacological effects: anti-inflammatory, antioxidant, antineoplastic, and hepatoprotective. The aim of this study was to protect *Plantago lanceolata* extract from hydrolysis and to improve its antioxidant effect using self-nano-emulsifying drug delivery systems (SNEDDS). Eight SNEDDS compositions were prepared, and their physical properties, in vitro cytotoxicity, and in vivo AST/ALT values were investigated. MTT cell viability assay was performed on Caco-2 cells. The well-diluted samples (200 to 1000-fold dilutions) proved to be non-cytotoxic. The acute administration of PL-SNEDDS compositions resulted in minor changes in hepatic markers (AST, ALT), except for compositions **4** and **8** due to their high Transcutol contents (80%). The non-toxic compositions showed a significant increase in free radical scavenger activity measured by the DPPH test compared to the blank SNEDDS. An indirect dissolution test was performed, based on the result of the DPPH antioxidant assay; the dissolution profiles of *Plantago lancolata* extract were statistically different from each SNEDDS. The anti-inflammatory effect of PL-SNEDDS compositions was confirmed by the ear inflammation test. For the complete examination period, all compositions decreased ear edema as compared to the positive (untreated) control. It can be concluded that PL-SNEDDS compositions could be used to deliver active natural compounds in a stable, efficient, and safe manner.

## 1. Introduction

*Plantago* is one of the most important genus within the Plantaginaceae family containing more than 200 species all around the world [1] *Plantago lanceolata* L. (*Ribwort plantain*) possesses various pharmacological properties for human health including antioxidant [2], anti-inflammatory [3], antineoplastic [4], hepatoprotective [5], immunoregulation, and neuroprotective properties, with an excellent and well-known safety profile. *P. lanceolata* has been also used in traditional medicine for its wound healing [6]. Its main active pharmaceutical ingredients belong to the group of phenylethanoid glycosides and iridoid glycosides [7]. Verbascoside (acteoside) is a phenylpropanoid glycoside and represents the main bioactive component of *P. lanceolata* [8]. The limitation of their formulations is their poor chemical stability due to hydrolysis [9]. The high hydrophilic character of verbascoside limits the range of possible applications. Stability studies have been reported about a greater stability of verbascoside in an O/W emulsion [10]. Attention has been focused on the development of Self-Micro-Emulsifying Drug Delivery Systems (SMEDDS) in order to stabilize herbal drugs and to enhance their bioavailabilities [11]. Micro- and nanosized drug delivery systems containing herbal drugs have been considered as ideal carrier systems for the optimization of the activity/efficacy of these extracts and for the management of problems that have emerged that are associated with plant [12]. SNEDDS is frequently used for the stabilization of natural products and these carrier systems may also increase the bioavailability of natural bioactive materials [13]. *P. lanceolata* is one of the best–characterized and described Plantaginaceae species, which is official in the European Pharmacopoiea [14]. It has been reported as a safe and effective herb, but few toxicological studies have been established/carried out about the biocompatibility of PL herbal extracts in different carrier systems. The application of high doses or the exposure for long periods may result in mutagenic and cytotoxic effects [1]. Microemulsions and SNEDDS often require a high content of surfactants, which can lead to skin or mucosal irritation. Amphiphilic molecules can be ideal surfactants and co-surfactants in SNEDDS, but toxic effects must be screened to prove their harmlessness [15].

This study was conducted to develop SNEDDS containing *Plantago lanceolata* extract (PL-SNEDDS), to improve the physical stability of the herbal extract, and to increase its antiinflammatory and antioxidant effect. The components of PL-SNEDDS were Isopropyl-myristate, Transcutol, and Labrasol/Kolliphor RH 40. Ternary phase diagrams demonstrated the existing zones of nanoemulsions. Nevertheless, toxicity studies (MTT-viability test on Caco-2 cells, investigation of liver enzymes in mice) were also performed to certify the safety profile of developed formulations. The ear inflammation test and DPPH antioxidant test verified the effectiveness of SNEDDS compositions and verified the improved bioavailability of investigated formulations.

## 2. Results

### 2.1. Bioactive Compounds in Plantago lancolata Leaves

The concentrations of bioactive compounds found in the Plantaginis lanceolatae folium are presented in Table 1. 10 mg/mL dry MeOH extract is equalent to 39.68 mg DW mL/mL plant drug. Acteoside is more hydrophilic than typical drug-like molecules (predicted logP = 0.82, logD = 0.81 at pH 7.50). Iridoid glycosides are even more polar (predicted logP = 3.18 and = −3.43 for aucubin and catalpol, respectively).

### 2.2. Formulation and Evaluation of Self-Nano-Emulsifying Drug Delivery Systems

**Figure 1:**
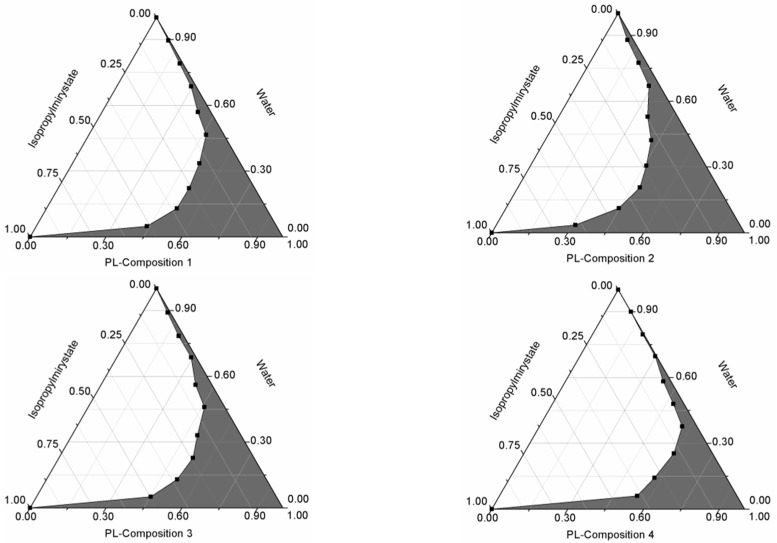

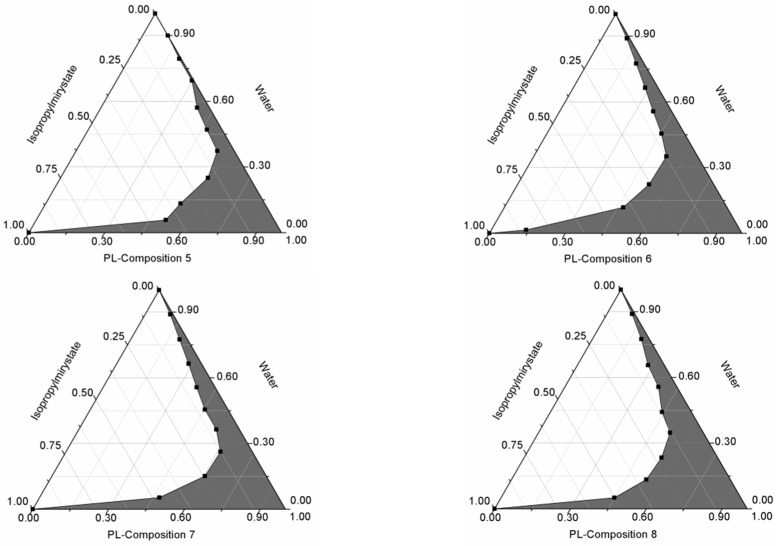
Pseudoternary phase diagrams of compositions **1**–**8**. (Shaded areas represented nanoemulsions).

**Figure 2:**
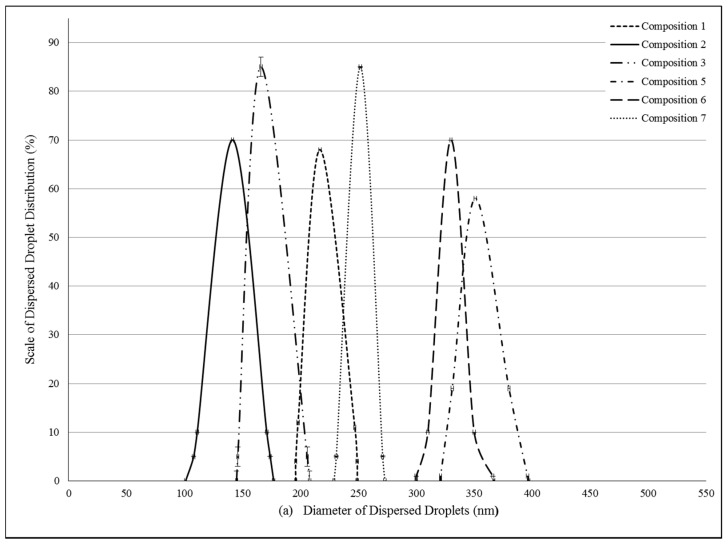

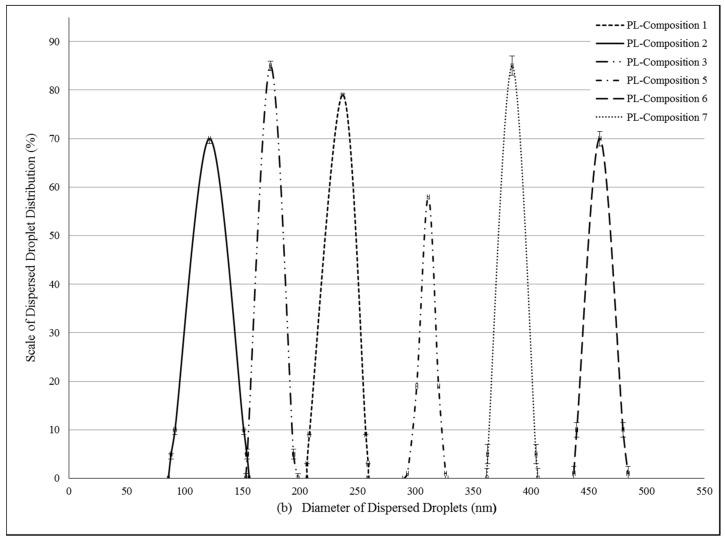
(**a**) Evaluated droplet size of self-nano-emulsifying drug delivery systems (SNEDDS) in water via dynamic light scattering (DLS) measurement. Evaluated average droplet sizes: composition **1**: 233.62 ± 2.34 nm, composition **2**: 141.51 ± 1.25 nm, composition **3**: 166.44 ± 1.05 nm, composition **5**: 374.89 ± 0.23 nm, composition **6**: 330.28 ± 0.95 nm, composition **7**: 251.81 ± 1.95 nm. Values are expressed as means ± SD, *n* = 5; (**b**) Evaluated droplet size of self-nano-emulsifying drug delivery systems (SNEDDS) in water via dynamic light scattering (DLS) measurement. Evaluated average droplet sizes: PL-composition **1**: 245.32 ± 0.64 nm, PL-composition **2**: 121.75 ± 1.00 nm, PL-composition **3**: 174.50 ± 1.00 nm, PL-composition **5**: 313.02 ± 3.12 nm, PL-composition **6**: 459.50 ± 3.85 nm, PL-composition **7**: 383.20 ± 2.55 nm. Values are expressed as means ± SD, *n* = 5.

Pseudoternary phase diagrams were constructed by using a conventional water titration technique. Isopropyl-myristate as oily phase, Labrasol or Kolliphor RH 40 as surfactant, and Transcutol HP as co-tenside were used in our compositions. The maximum nanoemulsion existing zones observed are shown in Figure 1. The distribution of droplet size is determined by dynamic light scattering (DLS) and presented in Figure 2. The developed SNEDDS-PL (shown in Table 2) spontaneously formed from nanoemulsion upon mild agitation in distillated water at room temperature. The concentration of *P. lanceolata* extract was 10 mg/mL in each composition. The investigated SNEDDS were stable for one month at room temperature. The screening of SNEDDS also involved the determination of the percentage of transmittance and the refractive index. The percentage of transmittance and refractive index of the resulted formulation were found to be 976.8 ± 1.31% and 1.337 ± 0.13%, indicating the transparency of these formulations. Based on the result of these experiments, eight compositions were selected for further investigations.

### 2.3. Stability Studies of Self-Nano-Emulsifying Drug Delivery Systems

The level *P. lanceolata* extract in SNEDDS remained more than 90% at day 10 in condition of 40 °C; relative humidity was 92.5%. While the temperature was increased to 60 °C, the level of herbal drug extract was decreased to 91.6%. These data suggested that SNEDDS is stable under the condition of high temperature and high humidity. Glycosides of Plantago species have a pH-dependent stability. While iridoids spontaneously decompose at very low pH (base of the Trim-Hill assay), acteoside becomes unstable at pH 7 and above. The original pH of the mixture (typically around pH 5–6) maintains stability of major compounds to an acceptable extent. This means that the extraction environment is “buffered” by the plant components and hence preserves most compounds during liquid extraction. The decomposing enzyme of iridoid glycosides (beta-glucosidase) is unable to operate in MeOH and, additionally, is inactivated by the heat.

### 2.4. Toxicity Investigations

#### 2.4.1. MTT Viability Assay on Caco-2 Cell Monolayers

**Figure 3:**
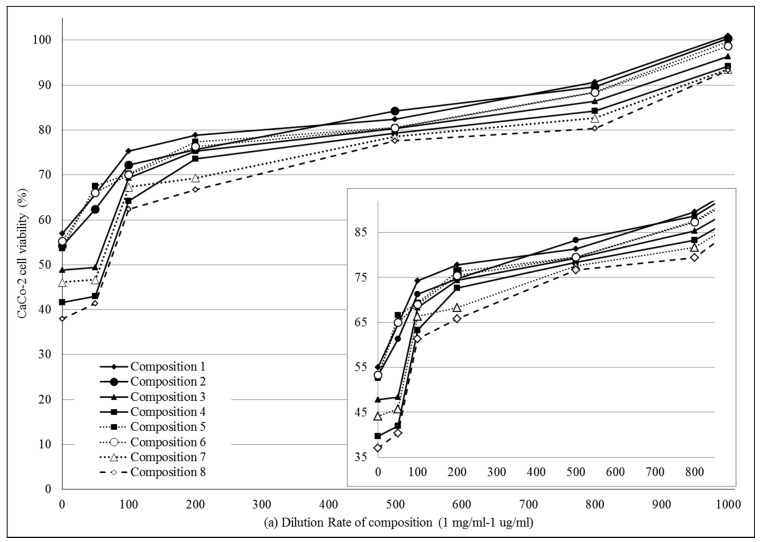

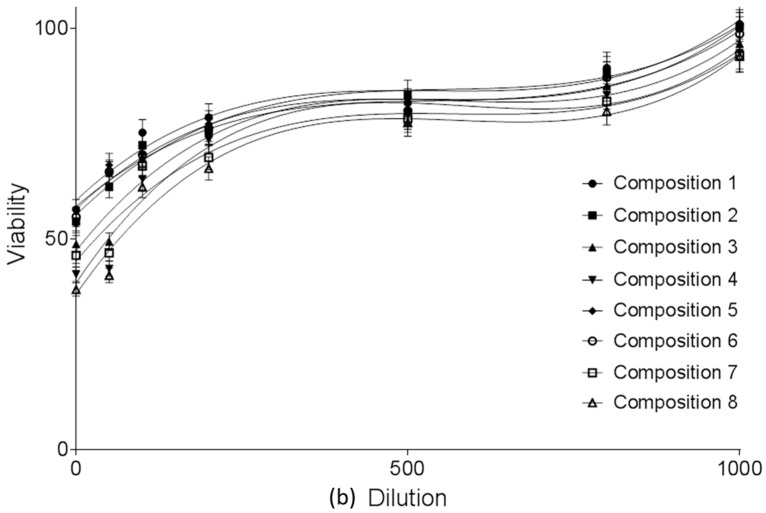
(**a**) Cell viability evaluation following MTT assay on Caco-2 cells treated with compositions **1**–**8** in the function of dilution ratio. 1 mg PL-SNEDDS composition was diluted by 1–1000 mL HBSS. Each data point represents the mean ± S.D., *n* = 10. Significant differences between groups for compositions **1**, **2**, **5**, **6** and groups for compositions **3**, **4**, **7**, **8** are marked with an asterisk (with the exception of composition **3** vs. compositions **5** and **6** at 100 dilution ratio, where the differences did not reach level of statistical significance). Positive control was Triton-X-treated group, negative control was Hank’s balanced solution (HBSS)-treated group; (**b**) Cell viability evaluation following MTT assay on Caco-2 cells treated with compositions **1**–**8** in the function of dilution ratio. 1 mg PL-SNEDDS composition was diluted by 1–1000 mL HBSS. Each data point represents the mean ± S.D., *n* = 10. Significant differences between groups for compositions **1**, **2**, **5**, **6** and groups for compositions **3**, **4**, **7**, **8** are marked with an asterisk (with the exception of composition **3** vs. compositions **5** and **6** at 100 dilution ratio, where the differences did not reach level of statistical significance). Positive control was Triton-X-treated group, negative control was Hank’s balanced solution (HBSS)-treated group. Linear regression of cell viability curves presented in the Figure 3a (regression parameters and goodness of fit data shown in Table 3).

MTT cell viability assay was performed on Caco-2 cells. (Figure 3a,b) Compositions **1**–**8** were diluted by Hank’s balanced solution (HBSS). 1 mg PL-SNEDDS composition was diluted by 1–1000 mL HBSS. There was a linear relationship between the cytotoxicity and the ratio of dilution of different SNEDDS compositions. The more concentrated samples decreased the cell viability and resulted in significant cytotoxicity. The higher the ratio of dilution of SNEDDS compositions, the better the cell viability of the Caco-2 cell line was. Application of those compositions (compositions **1**, **2** and **5**, **6**), in which the content of Transcutol HP was lower, resulted in higher cell viability values than in the cases of compositions **3**, **4** and **7**, **8**, where the range of Transcutol content was 60–80%. Nevertheless, it can be observed that there is a reduced cytotoxicity at a dilution of 200–1000. Significant differences between groups for compositions **1**, **2**, **5**, **6** and groups for compositions **3**, **4**, **7**, **8** (with the exception of composition **3** vs. compositions **5** and **6** at 100 dilution ratio, where the differences did not reach level of statistical significance). There are significant differences between PL-Composition-treated groups and positive or negative controls. After regression analysis, it seems that there are significant differences between groups for compositions **1**, **2**, **5**, **6** and groups for compositions **3**, **4**, **7**, **8** (Table 3).

#### 2.4.2. Effect of PL-SNEDDS on Hepatic Function Markers

Effect of all SNEDDS compositions containing *P. lanceolata* on hepatic function markers was investigated. Table 4 shows the effect of orally administrated SNEDDS-PL on liver function enzymes AST and ALT after 2 days of treatment. Composition **4** and **8**, containing 80% of Transcutol HP, resulted in fatal consequence, and number of dead mice were 5/5 and 4/5, respectively. These samples were removed from our experimental design. However, those compositions (SNEDDS-PL **1**, **2**, **3** and **5**, **6**, **7**), in which the range of Transcutol HP content altered between 33–60%, did not show significant increase in AST. Significant decrease of AST enzyme levels were observed. However, the changing of ALT enzyme level was not significant in each case. Moderate rise in ALT was recorded as compared with the normal control group. The results revealed that our samples excluding Compositions **4** and **8** may be safely applicable in our further experiments.

### 2.5. In Vitro Dissolution Study

**Figure 4:**
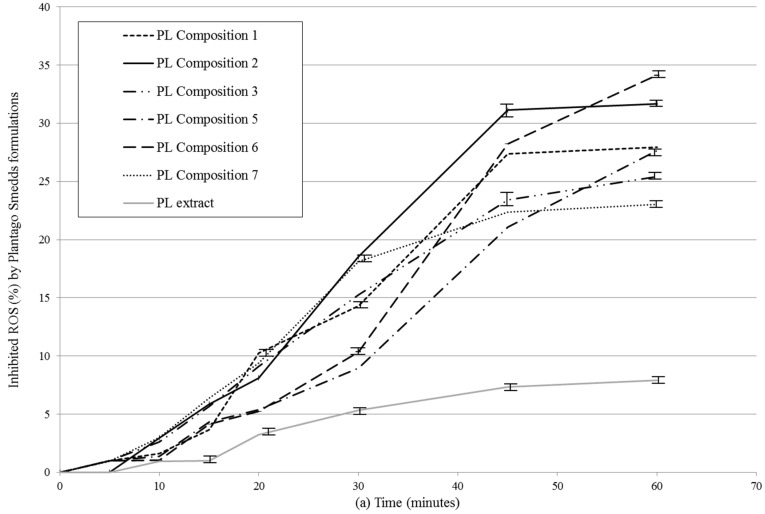

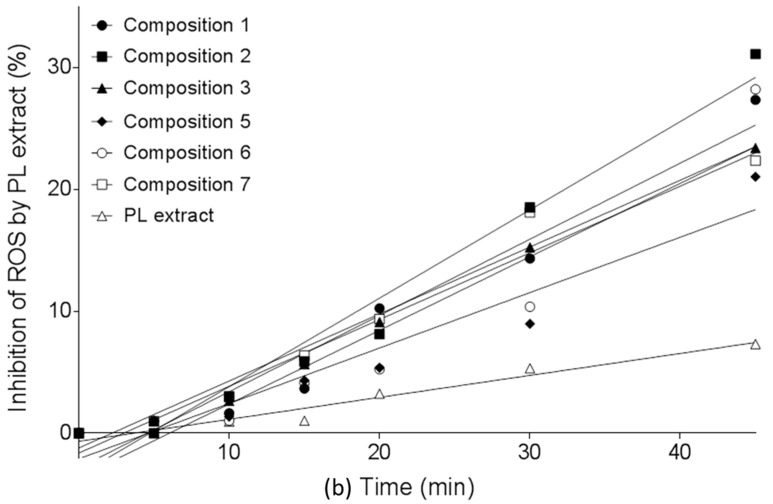
(**a**) In vitro dissolution study of PL-SNEDDS compositions based on the determination of DPPH (2,2-diphenyl-1-picrylhydrazyl) radical scavenging activity. Values are expressed as means ± SD, *n* = 5. Negative control was *Plantago lanceolata* extract without SNEDDS. All PL-composition-treated groups differed significantly from the negative control group (no mark is shown); (**b**) In vitro dissolution study of PL-SNEDDS compositions based on the determination of DPPH (2,2-diphenyl-1-picrylhydrazyl) radical scavenging activity. Values are expressed as means ± SD, *n* = 5. Negative control was *Plantago lanceolata* extract without SNEDDS. All PL-composition-treated groups differed significantly from the negative control group (no mark is shown). Linear regression of inhibition curves presented in Figure 4a. A (regression parameters and goodness of fit data shown in Table 5). As reactive oxidative species (ROS) inhibition data belonging to 60 min obviously deviate from linearity (to the same direction), they were omitted from this analysis.

In vitro release profiles of SNEDDS were investigated by the determination of antioxidant capacity of diffused samples. (Figure 4a,b, Table 5). Negative control was Plantago lanceolate extract without SNEDDS. All PL-Composition-treated groups differed significantly from the negative control group (no mark is shown).The DPPH inhibition of dissolved herb extract was significantly different from 15 min to 60 min. After 15 min, when the hard gelatin capsules were disintegrated, the SNEDDS formed nanoemulsion droplets rapidly and the antioxidant capacity of PL-Compositions was sharply increased. The antioxidant abilities of PL-Composition **2** and **6** were significantly higher than the other PL-Compositions. Based on the the DPPH antioxidant assay, the dissolution profiles of *P. lancolata* extract were statistically different from each SNEDDS. The efficacy of diffusion of PL-SNEDDS compositions were **4**–**5** fold higher than the extract alone.

### 2.6. DPPH Radical Scavenging Activity of SNEDDS-PL Samples

The DPPH antioxidant assay is based on the ability of a stable free radical (DPPH) to change color in the presence of antioxidants. In our antioxidant capacity measurements, compositions **1**–**3** and **5**–**7**, with or without *P. lanceolata* extracts, were tested. As controls the appropriate blank compositions (compositions **1**–**3** and **5**–**7**) were applied. Each sample contained 10 mg/mL *P. lanceolata* extract (PL-composition). The percentage of antioxidant activity (AA %) of each substance was assessed by DPPH free radical assay. The measurement of the DPPH radical scavenging activity was performed according to methodology described by Brand-Williams et al. [16]. There were significant differences between PL-composition treated groups and compositions without PL and PL-composition treated groups and PL-E treated sample (as positive control). These significant differences between PL-composition groups and the positive control group have been marked with an asterisk.

It has been demonstrated that all compositions were able to significantly inhibit DPPH mean oxidation. From PL-compositions, PL-composition **6** showed the most effective antioxidant activity in the DPPH assay. According to our measurements, PL-composition **7** showed the smallest activity, although its antioxidant capacity was found to be significantly higher than non-formulated *P. lanceolata* extract (PL-E).

### 2.7. Dimethyl-Benzene-Induced Ear Edema

**Figure 6:**
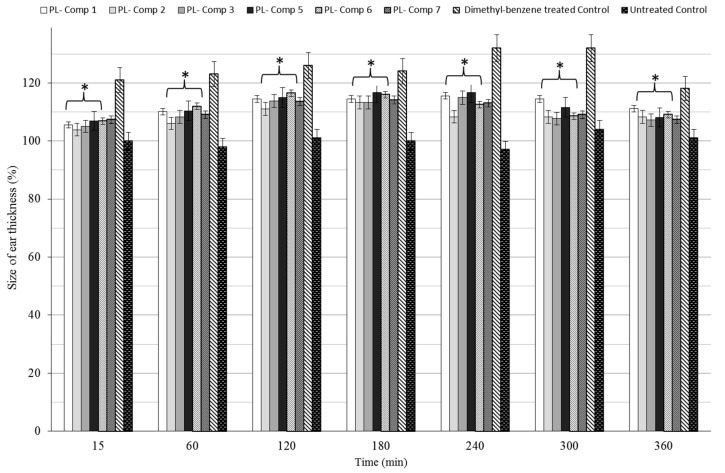
Time revolution of baseline corrected ear thickness (µm) in terms of different SNEDDS compositions containing 10 mg/mL *P. lanceolata* extract in a dimethyl-benzene-induced ear edema model in mice. The administered extract dose was 150 mg/kg/day formulated in SNEDDS and administered by gavage. For the complete time period, compositions showed significant differences to the positive control. As positive control, mice were treated with dimethyl-benzene without PL-SNEDDS pre-treatment, while in the negative control group, the mice received no treatment. Values are expressed as means ± SD, *n* = 6. All groups receiving a treatment with PL-components **1**–**3**, **5**–**7** differed significantly from their positive control group. Asterisk shown these significant differences.

Every orally-administered composition containing 10 mg/mL *P. lanceolata* extract significantly decreased ear thickness in the complete time period compared to positive control (dimethyl-benzene only), as seen in Figure 6. The maximum effect of dimethyl-benzene was measured at 2 h post-challenge time in either case. For the complete examination period, all compositions decrease ear edema as compared to the positive (untreated) control.

## 3. Discussion

*P. lanceolata* is a well-known species which exhibits various pharmacological effects [17]. However, the high capability of its bioactive components for hydrolysis resulted in poor stability of this natural extract [18]. Vertuani et al. [10] found 90% decrease of acteosid level at 40 °C and pH = 7, and 80% at pH = 6. In our study, we showed that self-nano-emulsifying drug delivery systems (SNEDDS) can be feasible solution to improve the stability of active pharmaceutical ingredients (APIs) of *P. lanceolata* extract. Liposomes for parenteral administration can also ameliorate the stability of verbascoside by preventing its hydrolysis [9]. Nevertheless, nanoemulsions may increase the stability of natural compounds and encapsulation improves the antioxidant activity of APIs [19]. It is worth noting that our PL-SNEDDS compositions potentiated the free radical scavenging activity of *P. lanceolata* extract compared to positive control (non-encapsulated *P. lanceolata* extract). DPPH reduction assay was an efficient and rapid method to screen the scavenger activity of PL-SNEDDS samples in vitro [20]. DPPH reaction is predictive but not sufficient to certify the therapeutic potential of *P. lanceolata* extract [19]. Therefore, ear inflammation test was carried out. Dimethyl-benzene is capable of inducing ear edema in the proper dose, as described earlier [21]; every PL-SNEDDS composition was able to decrease the dimethyl-benzene-induced inflammation. The *n*-hexane insoluble fraction of Plantago lanceolate demonstrated anti-inflammatory activities in mice. The fraction reduced the volume of paw edema and COX-2 expression as well [22].

Based on the predictive value of DPPH test, indirect dissolution test was developed. Those PL-SNEDDS compositions, which contain 25–25% Isopropyl-myristate—Kolliphor RH 40/Labrasol and 50% Transcutol HP, resulted in higher reduction of free radicals. According to the result of dissolution and ear inflammation tests, the ranking of PL-SNEDDS compositions was the same in the case of both tests. It is supported by Li et al. [23] experiment that stable SNEDDS-persimmon leaf extract formulations were prepared and linear in vitro-in vivo correlation was observed. Lipid-based nanosystems are also enable to increase the bioavailability of APIs, and hence more effective formulations can be developed [24]. The degree of efficacy depends on the types of oil, surfactant, and co-surfactant in these nano-emulsifying systems [25]. In our formulation, Transcutol HP as co-tenside was an effective penetration enhancer [26], isopropyl-myristate, as the oily phase, could dissolve the more lipophilic components of *P. lanceolata* [27], and Kolliphor RH 40/Labrasol was able to enhance the paracellular permeability of APIs on Caco-2 cell monolayer and was suitable to stabilize the most hydrophilic component of natural compounds [15,28]. Amphiphilic molecules can be ideal surfactants and co-surfactants in microemulsions [25], but cytocompatibility screening can support assess their toxicity profile. For this reason, in vitro MTT cell viability test was made and in vivo AST/ALT values were investigated in mice. In acute toxicity study the diluted PL-SNEDDS compositions was more concentrated than in cytotoxicity experiment on Caco-2 cells. However, the higher was the dilution, the safer were the compositions. The well diluted samples (200 to 1000-folds dilution) proved to be non-cytotoxic. These data are important to assess the toxicity profile of our compositions, but there are some limitations of investigations. Caco-2 cells are used extensively as an in vitro model for the rapid screening of intestinal absorption and cytotoxicity [29,30]. In vitro assay can predict irritancy, potential, and delayed toxicity of surfactants [31], but if it were complemented with different in vivo tests (i.e., determination of hepatic function markers, i.e., in animal experiments) then it could be more predictive. The toxicity ranking of PL-SNEDDS compositions was the same in both tests except for PL-SNEDDS compositions **4** and **8**. Because these samples resulted in the death of mice. It might indicate that the MTT cytotoxicity test is not appropriate to assess overall toxicity. More than one assay on different cell lines complemented with in vivo animal toxicity studies are applicable to determine the toxicity profile of compositions. Nevertheless, careful evaluation of in vitro and in vivo results are also needed to estimate the risk factors and possible outcomes in case of human exposure (parallelogram approach by Xing et al., 2006) [32,33].

## 4. Materials and Methods

### 4.1. Preparation and Characterization of Dry Plantago lanceolata Leaf Methanolic Extract

*P. lanceolata* leaves of pharmacopoeial quality were of commercial origin. It was reduced to a fine powder prior to further work. The powder was extracted with MeOH under reflux (100 g Dry Weight DW—400 mL MeOH) for 30 min, filtered, and evaporated to dryness in a rotary evaporator. Subsequently, the plant extract was defatted with hexane (3 × 50 mL) and dried again. 100 g plant drug yielded 25.2 g dry extract.

10 mg/mL solutions of the dry extract were prepared with MeOH and diluted 100 to 250-fold for analysis. Authentic standards of catalpol, aucubin, and acteoside were used as standards to construct the calibration curves in the concentration range 0.2–20 µg mL^−1^ (MeOH). The quantification of natural products by LC-MS was run on a Thermo Accela HPLC attached to a Thermo LTQ XL Linear Ion Trap MS (column: kinetex XB-C_18_ 100 mm × 2.1 mm × 2.6 μm). Gradient components were A, water with 0.1% (*v*/*v*) formic acid; B, acetonitrile with 0.1% (*v*/*v*) formic acid. The time programme was 5% B: 0–1 min, 5–20% B: 1–5 min, 20–60% B: 5-9 min, 60–100%: 9–11 min; 100% B: 11–13 min; 100–5% B: 13–15 min, 5% B: 15–17.5 min. Flow rate was 250 μL/min. Injection volume was 1 μL. Iridoids were detected in positive mode, and acteoside was detected in negative ion mode. Electrospray ionisation (ESI) parameters were as follows: heater temperature, unheated; sheath gas, N_2_; flow rate, 8 arbitrary units (arb); aux gas flow rate, 0 arb; spray voltage, 5 kV; capillary temperature, 275 °C. Capillary voltage was 7 V and −35 V in positive ion mode and negative ion mode, respectively. Calibration curves of 0.2–20 µg mL^−1^ were used, and pure compounds were dissolved in MeOH.

### 4.2. Preparation and Characterization of Dry Plantago lanceolata Leaf Methanolic Extract

*P. lanceolata* leaves of pharmacopoeial quality were of commercial origin. It was reduced to a fine powder prior to further work. The powder was extracted with MeOH under reflux (100 g DW—400 mL MeOH) for 30 min, filtered, and evaporated to dryness in a rotary evaporator. Subsequently, the plant extract was defatted with hexane (3 × 50 mL) and dried again. 100 g plant drug yielded 25.2 g dry extract.

10 mg/mL solutions of the dry extract were prepared with MeOH and diluted 100 to 250-fold for analysis. Authentic standards of catalpol, aucubin, and acteoside were used as standards to construct the calibration curves in the concentration range 0.2–20 µg mL^−1^ (MeOH).

### 4.3. Formulation and Evaluation of Self-Nano-Emulsifying Drug Delivery Systems

Different self-emulsifying combinations have been formulated by the water and oil dilution method with various previously tested tensides and co-tensides [15]. The compositions are listed in Table 1. Tenside components were mixed at 37 °C by Schott Tritronic dispenser (SI Analytical, Mainz, Germany) combined with Radelkis OP-912 magnetic stirrer (Radelkis, Budapest, Hungary). The applied concentrations of cytostatic drugs were dissolved in the systems at room temperature by permanent agitation. To evaluate any signs of phase separation, the mixtures were equilibrated for 24 h. An Erweka DT800 rotating paddle apparatus (Erweka GmbH, Heusenstamm, Germany) was used to evaluate the efficiency of self-emulsification of different mixtures. One gram of each mixture was added to 200 mL of distilled water with gentle agitation condition provided by a rotating paddle at 70 rpm and at a temperature of 37 °C. The process of self-emulsification was visually monitored for the rate of emulsification and for the appearance of the produced emulsions. The visual properties registered against the increment of the applied surfactant component in Ternary triangular diagrams. Plotting points of preferential combinations were selected according to cartesian coordinate calculation.

### 4.4. Determination of Droplet Size of PL-SNEDDS

The diameter of dispersed phase was investigated by a Cumulant dynamic light scattering (DLS) device (Malvern, Worchestershire, UK). The correlation of intensity function has been analyzed to obtain the diffusion coefficient. The measurements have been performed by a Brookhaven Fotometer Apparatus (Brookhaven, Upton, NY, USA). The operation temperature was adjusted to 25 °C, the laser detection angle to 90 degrees, Lambda to 533 nm, and the index to 1.334 by Particle Size Program 3.1 (Malvern, Worchestershire, UK). Diameters of dispersed droplets according to the diffusion coefficient have been evaluated automatically by the computer program.

### 4.5. Cell Culturing

Caco-2 (human adenocarcinoma cancer cells) was obtained from the European Collection of Cell Cultures (ECACC, Public Health England, Salisbury, UK). Cells were grown in plastic cell culture flasks in Dulbecco’s Modified Eagle’s Medium (Sigma-Aldrich Buchs, St. Gallen, Switzerland), supplemented with 3.7 g/L NaHCO_3_, 10% (*v*/*v*) heat-inactivated fetal bovine serum (FBS), 1% (*v*/*v*) non-essential amino acids solution, 1% (*v*/*v*) l-glutamine, 100 IU/mL penicillin, and 100 IU/mL streptomycin at 37 °C in an atmosphere of 5% CO_2_. The cells were routinely maintained by regular passaging. For cytotoxic and transport experiments, cells were used between passage numbers 20 and 40. The culture media was replaced with fresh media in every 72 h [33].

### 4.6. In Vitro Cell Viability Assay

To exclude any toxic effect of the blank SNEDDS and PL-SNEDDS on Caco-2 cells, MTT cell viability test was used [34]. Cells were seeded on flat bottom 96-well tissue culture plates at a density of 10^4^ cells/well and allowed to grow in a CO_2_ incubator at 37 °C for 4 days. For these studies, the culture medium was removed, surfactant or SNEDDS solutions were added, and the cells were incubated for a further 30 min. After removing the samples, another 3-h-incubation in a medium containing MTT at the concentration of 0.5 mg/mL followed. The dark blue formazan crystals were dissolved in acidic isopropanol (isopropanol:1.0 M hydrochloric acid = 25:1). The absorbance was measured at 570 nm against a 690 nm reference with FLUOstar OPTIMA Microplate Reader (BMG LABTECH, Offenburg, Germany). Cell viability was expressed as the percentage of the untreated control [35].

### 4.7. DPPH Radical Scavenging Activity of SNEDDS-PL Samples

Each sample (PL-Composition **1**–**3**, **5**–**8**, Composition **1**–**3**, **5**–**8**, PL-E) was reacted with the stable DPPH radical in ethanol (96%). The reaction mixture consisted of adding 100 µL of sample, 900 µL of absolute ethanol, and 2 mL of DPPH radical solution (0.06 mM) in absolute ethanol. The mixtures incubated for 30 min. When DPPH reacted with an antioxidant compound, which can donate hydrogen, it was reduced. The reaction resulted in color change from deep violet to light yellow. Quantitative measurement of remaining DPPH was carried out with an UV-spectrophotometer (Shimadzu Spectrophotometer, Tokyo, Japan) at a wavelength of λ = 517 nm. In case of photometric determination mixtures, absolute ethanol served as background. The control solutions were the same compositions without *P. lanceolata* extract. To demonstrate the improved antioxidant effect of combinations, blank *P. lanceolata* extract (10 mg/mL) was applied as well. The scavenging activity percentage (AA% = Antioxidant Activity) was determined according to Mensor et al. [35].

AA% = 100 − [((Abs_sample_ − Abs_blank_) × 100)/Abs_control_].

### 4.8. In Vitro Dissolution Test

In vitro dissolution test of PL-Compositions based on the determination of DPPH Radical Scavenging Activity of *P. lanceolata* extract.

In vitro drug release from PL-SNEDDS were conducted according to FDA-recommended dissolution methods in pH = 6.8. The dissolution condition was 500 mL of pH 6.8 phosphate buffer at a paddle speed of 75 rpm. Aliquots of 3 mL were withdrawn and filtered using 0.45 µm filter at predetermined time intervals of 5, 10, 15, 30, 60 min. The volume removed from each solution was replaced immediately with fresh dissolution medium. The determination of diffused *P. lanceolata* extract based on the DPPH Radical Scavenging Activity (see below).

### 4.9. Animals and Experimental Groups

Swiss male mice (22 ± 3 g), supplied by the Animal House of the School of Medicine, Faculty of Pharmacy, Ahvaz Jundishapur University of Medical Sciences, Ahvaz, Iran, (Ethical approval number is IR.AJUMS.REC. 1394.139) were used. The animals were maintained at a 12 h light/dark cycle, at constant temperature, with access to food and tap water ad libitum. All experimental procedures were approved by the Ethical Committee of Ahvaz Jandishapur University of Medical Sciences, Faculty of Pharmacy, Ahvaz, Iran. During the experiments, animals were processed according to the suggested international ethical guidelines for the care of laboratory animals. Fifty four animals were used for hepatic function markers investigations and forty-eight animals for ear oedema tests. For the first experiment, the mice were divided into nine groups (one control and eight groups for SMEDDS-PL investigation), for the second experiment, eight mice groups were composed (one positive and one negative control and six groups for SMEDDS-PL investigation). The extracts (150 mg/kg/day) were administered by gastric gavage (100 µL three times daily). The controls were given the same volume as in the test group.

The mice were anaesthetized on the final day of experiments, and blood was collected from venae cavae before mice were euthanized by cervical dislocation. 

### 4.10. Preparation of Blood Plasma

The collected blood was placed in heparinized tubes and centrifuged for 15 min at 2000 rpm in order to obtain plasma samples, which were used immediately to determine ALT and AST activities. 

### 4.11. Assay of Plasmatic Markers

The plasmatic activities of aspartate aminotransferase (AST) and alanine aminotransferase (ALT) were evaluated by the spectrophotometric method using commercially available kits (Roche reagents, Meylan, France), according to the manufacturer’s indication.

### 4.12. Dimethyl-Benzene-Induced Inflammation Model

Anasthesia was induced by thiopental in an amount of 50 mg/kg intraperitoneally (i.p.), repeated as required. The posterior area of the right ear was then injected with 3 m/m % dimethyl-benzene solution. This treatment was applied 30 min after the oral gavage. Thus, the oral administration of SNEDDS-PL was performed firstly, and the induction of inflammation was carried out secondly.

### 4.13. Measurement of Ear Oedema

Ear thickness was measured by a micrometer caliper (Oxford Precision, Leicester, UK), with 0.1 mm accuracy before dimethyl-benzene treatment and 15 min after the first dimethyl-benzene application, then by each hour during a 6 h period after each 3 m/m % dimethyl-benzene treatment according to Ujhelyi J., et al. [21]. SNEDDS-PL treatment was performed 30 min before starting time of ear edema induction. Data were expressed in micrometers.

### 4.14. Statistical Analysis

Data were handled and analyzed using Microsoft Excel 2013 and SigmaStat 4.0 (version 3.1; SPSS, Chicago, IL, USA, 2015), and herein presented as means ± SD. Comparison of results of MTT cell viability assays, hepatic function markers (AST, ALT), free radical scavenging activity test, in vitro dissolution test, and ear edema test was performed with one-way ANOVA and repeated-measures ANOVA followed by Tukey or Dunnett post testing. Difference of means was regarded as significant in case of *p* < 0.05. All experiments were carried out in quintuplicates and repeated at least five times (*n* = 5).

## 5. Conclusions

Finally, it can be concluded that PL-SNEDDS compositions have a high potential to improve the stability of APIs of *Plantago lanceolata* extract and also can improve the antioxidant and anti-inflammatory effect of natural compounds. These samples could be a feasible alternative for medical purposes.

Nevertheless, in vitro cytocompatibility and in vivo animal studies indicated that more tests are needed to estimate the overall toxicity profile of PL-SNEDDS compositions.

## Figures and Tables

**Figure 5:**
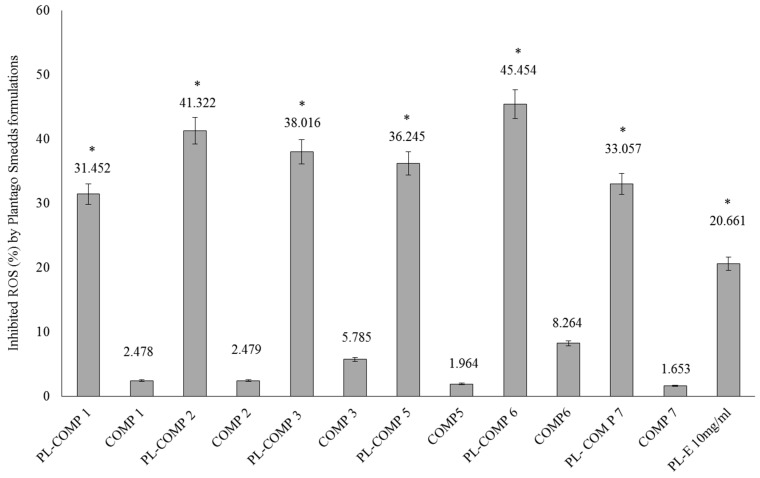
Inhibition of (ROS, %) by different PL-compositions. The positive control was *P. lanceolata* extract in a concentration 10 mg/mL. The negative controls were the compositions without *P. lanceolata* extract. Values are expressed as means ± SD, *n* = 5. Significant differences between PL-composition groups and the positive control group have been marked with an asterisk.

**Table 1:** Chemical description of Catalpol, Aucubin, and Acteoside in plantain (*Plantago lanceolata*).

	Catalpol (CA)	Aucubin (AU)	Acteoside/Verbescoside (ACTE)
Chemical structures of bioactive components	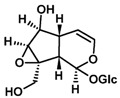	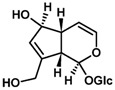	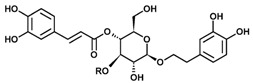
Content in MeOH extract	1.21 ± 0.02%	2.34 ± 0.01%	5.99 ± 0.012%

**Table 2:** Compositions of *Plantago lanceolata* extract (PL-SNEDDS) (**1**–**8)**. The components were Isopropyl myristate as oily phase, Labrasol or Kolliphor RH 40 as surfactant, and Transcutol HP as co-tenside. The concentration of *Plantago lanceolata* extract was 10 mg/mL in all samples.

Number of Compositions	Isopropyl-Myristate	Transcutol HP	Kolliphor RH 40	Labrasol
**1.**	33%	33%	33%	-
**2.**	25%	50%	25%	-
**3.**	15%	60%	15%	-
**4.**	10%	80%	10%	-
**5.**	33%	33%	-	33%
**6.**	25%	50%	-	25%
**7.**	15%	60%	-	15%
**8.**	10%	80%	-	10%

**Table 3:** Regression parameters using cuboid polynomial equation and goodness of fit data for the cell viability evaluation following MTT (3-(4,5-dimethylthiazol-2-yl)-2,5-diphenyltetrazolium bromide) assay on Caco-2 cells treated with compositions **1**–**8** (see: Figure 3, Panel B). SE = Standard Error. (Comp. = Composition).

	Comp. 1	Comp. 2	Comp. 3	Comp. 4	Comp. 5	Comp. 6	Comp. 7	Comp. 8
**B0**	59.16	55.65	47.21	39.53	57.13	57.72	44.76	36.85
**B1**	0.1483	0.1551	0.1984	0.2363	0.15	0.1353	0.187	0.2282
**B2**	−2.793 × 10^−4^	−2.73 × 10^−4^	−3.577 × 10^−4^	−4.222 × 10^−4^	−2.848 × 10^−4^	−2.477 × 10^−4^	−3.303 × 10^−4^	−4.08 × 10^−4^
**B3**	1.738 × 10^−7^	1.631 × 10^−7^	2.092 × 10^−7^	2.414 × 10^−7^	1.783 × 10^−7^	1.541 × 10^−7^	1.924 × 10^−7^	2.366 × 10^−7^
**B0 SE**	1.42	1.283	1.766	1.771	1.43	1.283	1.752	1.627
**B1 SE**	1.785 × 10^−2^	1.613 × 10^−2^	2.22 × 10^−2^	2.226 × 10^−2^	1.798 × 10^−2^	1.613 × 10^−2^	2.202 × 10^−2^	2.045 × 10^−2^
**B2 SE**	4.45 × 10^−5^	4.022 × 10^−5^	5.535 × 10^−5^	5.551 × 10^−5^	4.483 × 10^−5^	4.022 × 10^−5^	5.49 × 10^−5^	5.1 × 10^−5^
**B3 SE**	2.916 × 10^−8^	2.635 × 10^−8^	3.627 × 10^−8^	3.637 × 10^−8^	2.937 × 10^−8^	2.635 × 10^−8^	3.597 × 10^−8^	3.341 × 10^−8^
**R^2^**	0.9296	0.9501	0.9252	0.9399	0.9301	0.94	0.925	0.9502

**Table 4:** Effect of SNEDDS containing *P. lanceolata* extract on serum AST and ALT enzyme activities. Each value represents the means ± SD for 6 mice and is expressed in IU/L or U/L. Groups treated with the compositions **1**–**8** were compared to the control group, significant differences were marked with an asterisk. Administration of compositions **4** and **8** resulted in the death of mice within 2 days (ND = No Data), (Comp. = Composition).

	Control	Comp. 1	Comp. 2	Comp. 3	Comp. 4	Comp. 5	Comp. 6	Comp. 7	Comp. 8
AST (IU/L)	362.28 ± 12.3	183.6 ± 34 *	225.78 ± 13.4 *	285.90 ± 12.3 *	ND	232.56 ± 10.6 *	214.6 ± 12.3 *	298.78 ± 33.2 *	ND
ALT (U/L)	92.34 ± 23.4	113.23 ± 42.1	98.70 ± 29.7	145.62 ± 38.6 *	ND	93.4 ± 21.4	119.21 ± 13.2	138.23 ± 12.6 *	ND

**Table 5:** Regression parameters and goodness of fit data for the in vitro dissolution study of PL-SNEDDS compositions based on the determination of DPPH radical scavenging activity (see: Figure 5b). SE = Standard Error, (Comp. = Composition).

	Comp. 1	Comp. 2	Comp. 3	Comp. 5	Comp. 6	Comp. 7	PL Extract
Slope	0.625	0.725	0.548	0.454	0.604	0.549	0.18
(±SE)	(±0.061)	(±0.061)	(±0.027)	(±0.056)	(±0.098)	(±0.043)	(±0.017)
Y-intercept	−2.849	−3.436	−1.632	−2.108	−3.635	−1.213	−0.662
(±SE)	(±1.408)	(±1.415)	(±0.627)	(±1.292)	(±2.235)	(±0.988)	(±0.382)
X-intercept	4.556	4.737	2.979	4.638	6.023	2.207	3.677
R^2^	0.954	0.9651	0.9877	0.9286	0.8844	0.9702	0.9588
